# Sow reproductive disorders: a key issue affecting the pig industry

**DOI:** 10.3389/fvets.2025.1535719

**Published:** 2025-03-05

**Authors:** Yihan Wang, Youshun Jin, Yanyan Wang, Yunhui Li, Xiaoxue Wang, Zhaocai Li, Jizhang Zhou

**Affiliations:** ^1^State Key Laboratory for Animal Disease Control and Prevention, Lanzhou Veterinary Research Institute, Chinese Academy of Agricultural Sciences, College of Veterinary Medicine, Lanzhou University, Lanzhou, China; ^2^Animal Pathology Laboratory, College of Veterinary Medicine, Northwest A&F University, Xianyang, China; ^3^College of Life Sciences, Yulin University, Yulin, China

**Keywords:** sows, reproductive disorders, abortion, vaccine, integrated management measures

## Abstract

Pig farming is essential to global agricultural economies and food security. However, reproductive disorders in sows significantly impact the economic viability and sustainability of the pig industry. These disorders often result from complex interactions between pathogenic and non-pathogenic factors. Preventing abortions is typically more cost-effective than managing and treating them, particularly in intensive pig farming system. This highlights the importance of comprehensively understanding the underlying causes of abortion in sows. This review explores the factors contributing to sow reproductive disorders, including both non-infectious factors (environmental conditions and management practices) and infectious factors (viruses, bacteria, and parasites). We also outline preventive and control strategies, alongside integrated management approaches, by analyzing the underlying causes and pathogenic mechanisms of pregnancy disorders. Overall, implementing the “One Health” concept in large-scale farming provides an effective strategy to reduce the incidence of sow abortion rate, ensure stable livestock production, and maintain a reliable global pork supply.

## 1 Introduction

Pig production plays a significant role in global meat consumption, contributing 34% of the world’s meat supply (1). Over the last six decades, from 1962 to 2022, pork consumption has increased, leading to an impressive 130% increase in global pork production (2). By 2024, the global pig population reached 1.25 billion, with pork production reaching 114.20 million tons (3). China accounting for 54.0% of the world’s pig population (678.0 million heads), followed by the EU (232 million heads). Over the past decade, a key factor driving this growth has been significant improvements in reproductive performance, particularly advancements in the breeding and management of modern hybrid sows. These improvements have directly contributed to increase in the number of piglets weaned per sow per year (PWSY) (4, 5). However, reproductive disorders of sows seriously affect the economic and sustainable development of the pig farming industry.

**Figure 1:**
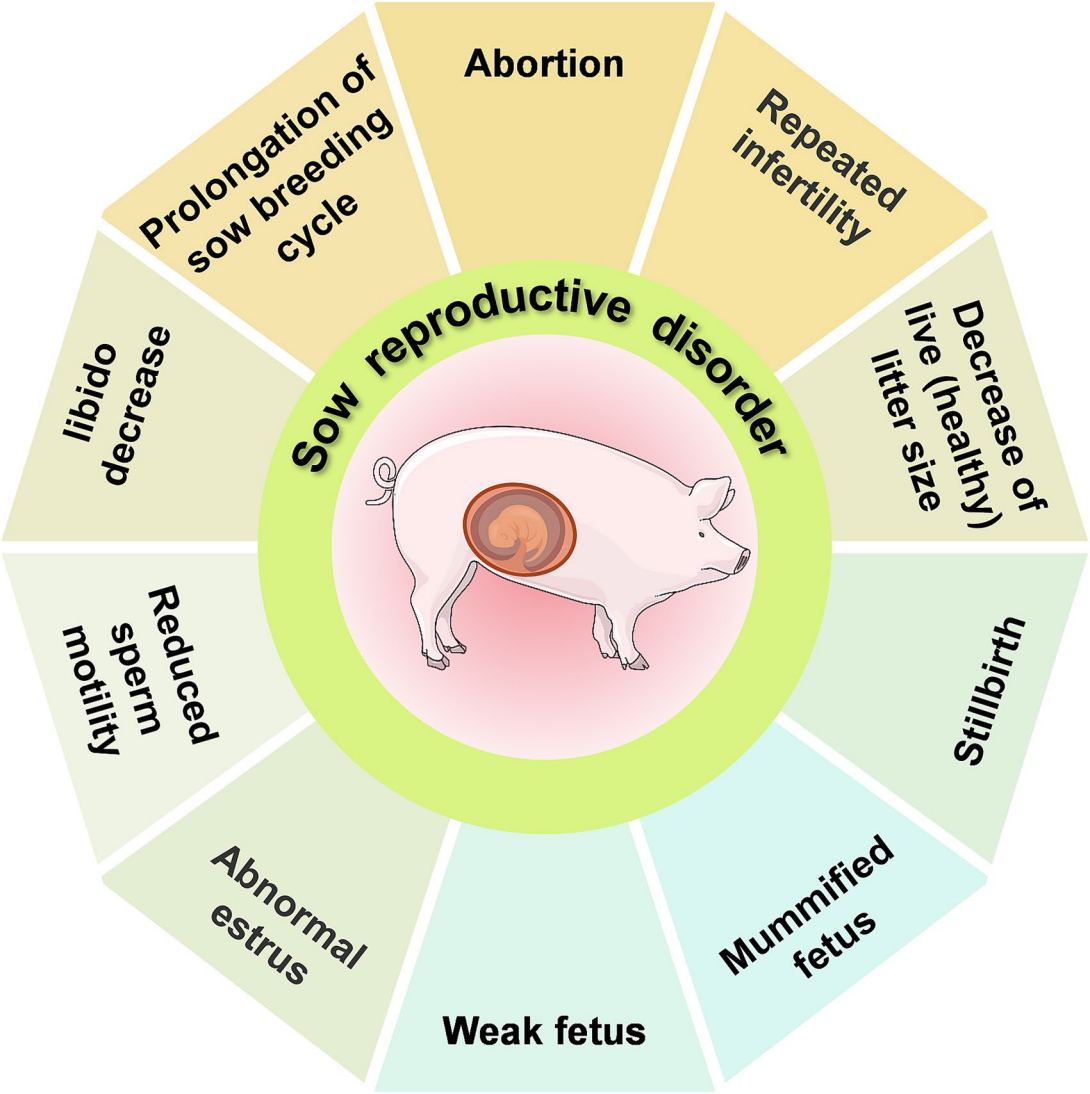
An overview of the SMEDI (stillbirth, mummification, embryonic death, and infertility) syndrome.

Reproductive disorders in sows exhibit various clinical manifestations. Among these, the SMEDI (stillbirth, mummification, embryonic death, and infertility) syndrome (Figure 1) displays a disturbed gestation in sows (6). Abortions in sows can be caused by a range of factors, both non-infectious and infectious. The main non-infectious causes are linked to external environmental conditions (temperature, humidity, and air quality) and feeding management practices (feed quality and reproductive feeding techniques), which can stress the animals and affect their reproductive performance. Infectious factors are caused serious threat to reproductive health in pigs, The main infectious factors include viral infections (e.g., porcine parvovirus, porcine pseudorabies, porcine reproductive and respiratory syndrome virus, Japanese encephalitis B virus, porcine circovirus, and classical swine fever virus), bacterial infections (e.g., brucellosis, listeriosis, chlamydiosis, leptospirosis, campylobacteriosis, and swine erysipelas), and parasitic infections (e.g., *Toxoplasma gondii*). The etiology of reproductive disorders in sows is not solely attributed to a single pathogen but often involves mixed infections of multiple pathogens. Non-infectious factors play a more significant role in sow reproductive disorders than that of infectious factors. However, reproductive disorders caused by non-infectious factors can be more effectively managed through changes in integrated management practices than infectious factors, which may pose a greater risk for epidemic outbreaks.

The One Health approach requires understanding the interactions between the pathogens that cause reproductive disorders in sows and other animal hosts, and considering the effects of external environmental conditions and management practices, to achieve One Health for all living organisms (including humans) on large farms. In this paper, we review the various non-infectious (seasonal, environmental, nutritional and mycotoxins) and infectious (viral, bacterial, and parasitic) factors associated with reproductive disorders in sows in terms of causes, pathogenesis and integrated management practices.

## 2 Non-infection causative factors of abortion

Non-infectious factors affecting the reproductive performance of sows account for more than 70.0% of abortions and fetal deaths in sows (7), with external environmental factors and management practices being the primary contributors. Temperature and humidity play a vital role in hormone secretion and overall reproductive health in sows. Specifically, high temperature and humidity can induce heat stress, which affects hormone secretion and can lead skin and limb diseases. Consequently, inappropriate rearing environments can disrupt the sow’s endocrine system, ultimately causing luteal regression and subsequent abortions (Table 1).

**Table 1:** The main non-infectious causes of abortion.

Factors	Effects		References
**Environment**			
Temperature	The optimum temperature for the first trimester is 13 ~ 19°C, that for the second trimester is 16–20°C		Robbins (118), Muns et al. (119)
	>28°C prolongs sexual maturity of sows; >30°C causes endocrine system imbalance in sows		Zhang et al. (120)
	Heat stress due to high temperatures (decreased estrogen secretion, abnormal estrus, affecting sow pregnancy rate, embryo attachment, embryo development, causing abortion and weak litter size increase, etc.)		Omtvedt et al. (121)
Humidity	The appropriate relative humidity for sow breeding is 60.0% ~ 70.0%		Ma et al. (122)
	Long-term high temperatures and high humidity environments can prolong the estrus interval of pregnant sows, significantly increase the weak litter rate and stillbirth rate of pregnant sows, and increase the return rate of first-time sows after breeding		
Air quality	High contents of toxic gasses such as ammonia (NH_3_), hydrogen sulfide (H_2_S), and carbon monoxide (CO) cause reproductive disorders in sows		Wenke et al. (123), Pejsak et al. (124)
**Nutrition**			
Energy	High energy levels result in fat deposition around the uterus and hinder follicle development, affecting fertilization and implantation		Yang et al. (125), Meng et al. (126)
	Insufficient energy intake during lactation impairs embryonic development, prolongs estrus intervals, and reduces fertility in sows after weaning		Fang et al. (127), Gu et al. (128)
Protein	Lack of proteins hinders the development of the reproductive system and delays the estrus		Fang et al. (127)
Vitamin	Insufficient vitamin E results in anorexia blocked sex hormone synthesis, and luteal degeneration		Pinelli-Saavedra (129)
	Vitamin A and vitamin D deficiencies may lead to immune imbalances that increase the risk of pregnancy loss		Al Balawi et al. (130), McCauley et al. (131)
Trace elements	Selenium (Se)	Selenium deficiency in sows results in elevated oxidative stress levels, which compromises their antioxidant defense system. This disruption can lead to reduced reproductive performance and may negatively affect fetal development through the placenta	Surai and Fisinin (132)
	Zinc (Zn)	Zinc deficiency results in stagnant ovarian development and impaired uterine epithelial development in sows, while in males, it induces testicular atrophy and reduces fertility	Duffy et al. (133), Liu et al. (134)
	Magnesium (Mg)	Magnesium deficiency may elevate stress and oxidative stress levels in sows, compromising immune function and reducing productivity. Additionally, it may impair embryo development, leading to poor outcomes or malformations	Zang et al. (135), Guo et al. (136), Halliwell et al. (137)
**Mycotoxins**			
Zearalenone	Amnesia, abortion, embryo implantation obstruction, fetal death, ovarian atrophy, etc.		Gao et al. (138), Zhou et al. (139)
Deoxynivalenol	Inhibition of oocyte maturation and embryonic development, resulting in decreased conception rates		Malekinejad et al. (9)
Ergot alkaloids	Growth arrest, abortion, reproductive interruption, agalactia, etc.		Waret-Szkuta et al. (10)
Aflatoxin B1	Non-estrus, repeated mating infertility, abortion, embryo arrest, inhibition of cell proliferation, etc.		Shin et al. (11)
T-2 Toxin	Infertility, ovarian tissue atrophy, induction of granulosa cell apoptosis, etc.		Yang et al. (125)
**Others**			
Seasonal infertility	During late summer or early autumn, the sow ovarian progesterone secretion declines, with reduced oocyte development leading to severe delay of embryo implantation or difficulty maintaining a pregnancy		Bertoldo et al. (140)
Stress	Hormonal changes, increased body temperature, and uterine contractions caused by excitement		Peltoniemi et al. (141), Einarsson et al. (142)
**Technological factors**			
Dock density	High-density feeding can lead to increased stress levels in sows, potentially affecting embryo survival and pregnancy success		Spoolder et al. (143)
Artificial insemination	Bacterial and fungal contamination of boar semen increases the infection risk in inseminated sows, thereby reducing their reproductive performance		Nitsche-Melkus et al. (15), Ciornei et al. (16)

In addition to environmental factors, feeding practices are critical determinant of sow reproductive capacity. Feed quality directly impacts conception rates and fetal development. Overnutrition can lead to obesity, thereby reducing conception rates, while malnutrition decreases reproductive hormone synthesis, impeding reproductive system development and delaying estrus. Notably, sows consuming moldy feed accumulate toxins that induce reproductive disorders. Research indicates that mycotoxins, such as Zearalenone (ZEN), exhibit estrogen-like activity, compete for receptors, inhibit follicle-stimulating hormone (FSH) secretion, and disrupt the endocrine system. T-2 toxin disrupts the reproductive endocrine axis and inhibits reproductive hormone synthesis. Deoxynivalenol (DON) inhibits oocyte maturation and embryonic development (8, 9). Ergot Alkaloids lead to agalactia in sows and to a high neonatal mortality rate (10). Aflatoxin B1 (AFB1) impairs oocyte maturation and damage early embryonic development through oxidative stress and mechanisms such as apoptosis and autophagy (11).

The impact of rearing and breeding techniques, such as stocking density and artificial insemination (AI), on sow reproductive performance should not be overlooked. In most major pork-producing countries, AI is highly efficient (12). However, Semen is an ideal medium for the establishment and growth of many microorganisms including bacteria and fungi (13). Consequently, during collection, semen is susceptible to contamination from sources such as boar feces, preputial secretions, and the environment in which it is collected and processed (14). Contamination of boar semen with bacteria (e.g., *E. coli*, *Pseudomonas* spp., *Staphylococcus* spp., *Proteus* spp.) and fungi (e.g., *Candida* spp., *Aspergillus* spp.) can reduce sperm viability and increase the risk of infection in inseminated sows, such as endometritis, ultimately reducing reproductive performance (15, 16).

## 3 Infection causative factors of abortion

Infectious factors have received more attention than non-infectious ones due to their association with epidemics of reproductive failure in sows (Table 2).

**Table 2:** Main pathogens involved in sow reproductive disorder.

Disease	Pathogens	Clinical symptom	Laboratory diagnostics	Control methods
**Viral**				
Porcine parvovirus infection	Porcine parvovirus, PPV	Abortion, stillbirths, mummification	PCR, ELISA	Vaccines
Porcine pseudorabies	Pseudorabies virus, PRV	Respiratory disease, acute neurological disease, abortion	PCR, ELISA	Vaccines
Porcine reproductive and respiratory syndrome	Porcine reproductive and respiratory syndrome virus, PRRSV	Mild depression, anorexia, fever, abortion, stillbirths, umbilical cord edema	PCR, IF	Vaccines
Japanese encephalitis B	Japanese encephalitis virus, JEV	Abortion, stillbirth, premature or delayed delivery, acute orchitis	RT-PCR, LAT	Vaccines
Porcine circovirus disease	Porcine circovirus, PCV	Abortion, stillborn, mummification, Congenital Tremors	PCR, IHC	Vaccines
Classical swine fever	Classical swine fever virus, CSFV	Fever, anorexia, depression, ataxia, cutaneous erythema	RT-PCR, FAVN	Vaccines
**Bacterial**				
Brucellosis	*Brucella suis*	Placentitis	Bacterial culture, rose bengal test	Whole-herd depopulation
Listeriosis	*Listeria monocytogenes*	Meningitis, septicemia, mononucleosis, abortion	Bacterial culture, PCR	Antibiotic
Chlamydiosis	*Chlamydia* spp.	Abortion, periparturient dysgalactiae syndrome, return to oestrus, mummification, delivery of weak piglets	Ag-ELISA, IHC, IHA	Antibiotic
Campylobacteriosis	*Campylobacter* spp.	enteritis, abortions and infertility in various species	IF, ELISA, PCR	Antibiotic
Swine erysipelas	*Erysipelothrix rhusiopathiae*	Fever, anorexia, depression, skin lesions	Bacterial culture, PCR	Vaccines
Leptospirosis	*Leptospira* spp.	Transient fever, anorexia, depression, Occasional fetal jaundice	PCR, MAT	Vaccines
**Parasitic**				
Toxoplasmosis	*Toxoplasma gondii*	Abortion	Serology	Biosecurity measures

### 3.1 Viral infections

#### 3.1.1 Porcine parvovirus

Porcine Parvovirus (PPV), an Ungulate parvovirus 1 in the *Protoparvirus* genus, was first recognized as a member of the *Parvoviridae* family and causative agent of SMEDI syndrome at the end of the 1960s (17). Seven distinct genotypes of PPV (PPV1-PPV7), which are prevalent worldwide, have been identified.

**Figure 2:**
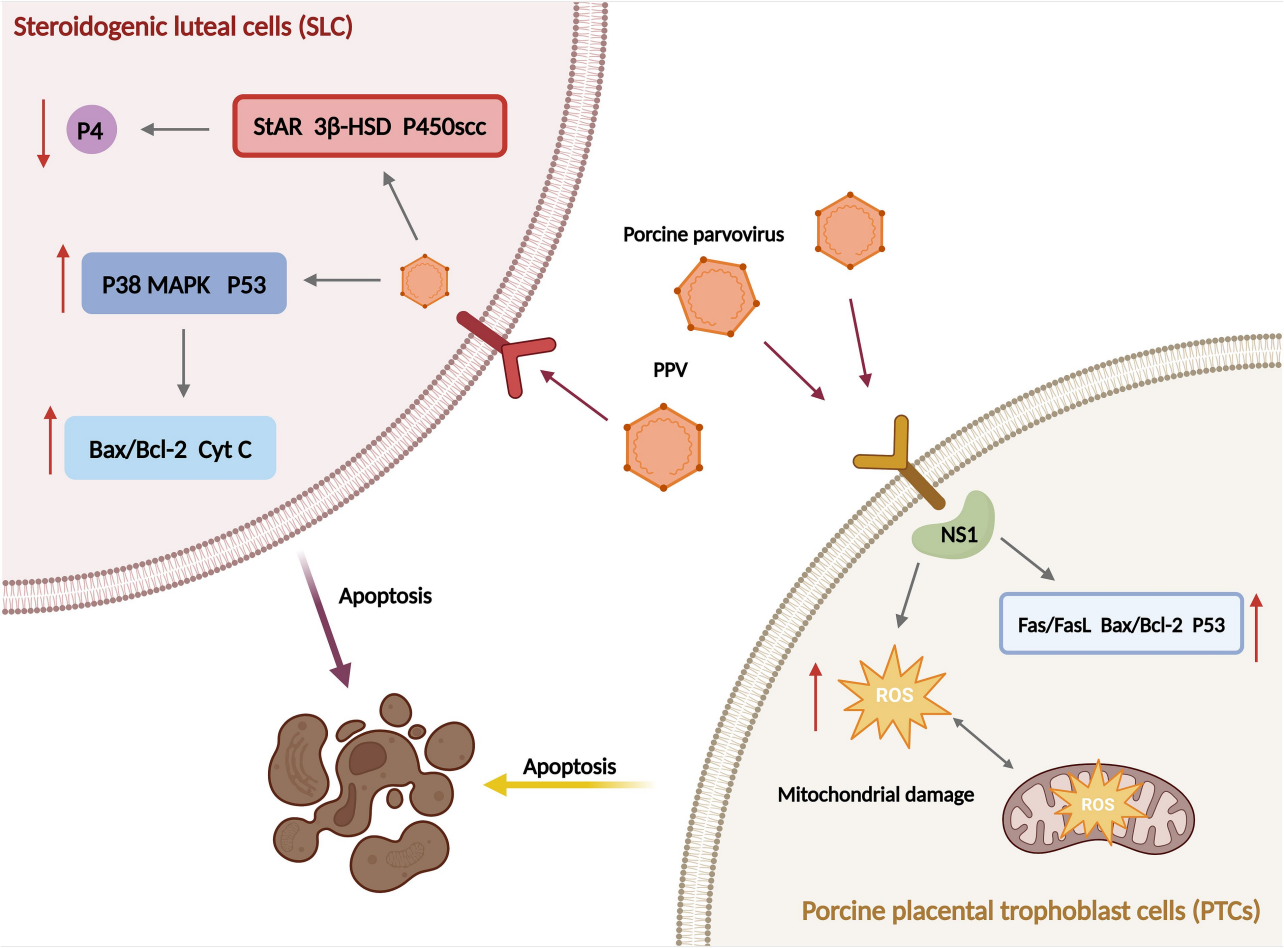
Mechanisms of reproductive dysfunction resulted from luteal cells and placental trophoblast cells apoptosis induced by porcine parvovirus (PPV) infection in sows.

The Ministry of Agriculture in China has classified PPV as a Class II animal disease pathogen (18). In China, the positivity rate of PPV was significantly higher in pigs in the south-west, northern and southern parts of the country. For instance, in Haikou and Chongqing, China (2014), the serological positivity rate of PPV reached over 90% (19), while 85% of pig herds with reproductive dysfunction syndrome was positive for PPV in Yunnan Province (20). In Pakistan, Punjab (2016), the seroprevalence of PPV was 41.1% (21). In recent years, there has been an increase in the PPV variation and its co-infection with other pathogens. An epidemiological survey of the porcine reproductive syndrome in South-west China (2012) showed that the positive rate of PPV was 43.97%, while that of Pseudorabies virus (PRV) was 24.6%, and *Chlamydia psittaci* (Cps) was 36.98%. Of these, 39.6% were mono-infections, while 35.6% were mixed infections (22). It has been shown that PPV infection-induced cell apoptosis in pregnant sows is primarily caused by the non-structural protein NS1. This process is characterized by the induction of host cell DNA damage, reactive oxygen species (ROS) generation and mitochondrial damage (23). A consequence of PPV infection is the nuclear fragmentation and subsequent nucleus consolidation of the luteal cells, which damages the luteal tissue of sows. Moreover, PPV impedes progesterone synthesis in luteal cells by inhibiting the expression of StAR, 3β-HSD and P450scc and induces apoptosis in luteal cells by activating the p38, p53 and mitochondrial pathways (Figure 2), culminating in abortion and infertility (24). Additionally, PPV induces apoptosis in embryonic trophoblasts by regulating the expression levels of Fas/Fas L, Bax/Bcl-2 and p53, ultimately resulting in embryonic death (25).

PPV has a single serotype, and vaccine immunization has become the primary prevention and control strategy for the pathogen. The most commonly used vaccines in clinical settings are live and inactivated weakly-attenuated vaccines.

#### 3.1.2 Porcine pseudorabies

Porcine pseudorabies (PR), also known as Aujeszky’s disease, is caused by the pseudorabies virus (PRV), which has a wide host range. The family Suidae (true pigs) are the natural hosts and reservoirs of PRV (26–28). There are two types of PRV infections: overt and latent. Adult pigs are mostly latently infected and can continuously excrete the virus (29). Following PRV infection in boars, the virus can be excreted in semen and transmitted to sows, leading to various reproductive disorders. Serological tests showed that the positivity rate of PRV gE antibody in the 3,449 serum samples collected from the Hebei Province, China (2022), was 46.27% (30). In Greece (2019), 28.6% of 42 selected pig farms were positive for antibodies against the wild-type strains of PRV (31).

PRV can enter the blood circulation via leukocyte uptake, allowing it to reach all body parts, including the placental tissues, where it can cause stillbirth or miscarriage following fetal invasion (32). PRV-infected mononuclear cells can cross the endothelial cell (EC) barrier of the maternal vasculature (33), and widespread EC infection can lead to detachment of membranes in early gestation, abortions of virus-negative fetuses, or fetal reabsorptions in the sow. Secondary replication in the EC of the uterus of pregnant sows can cause vasculitis and multifocal thrombosis, and microscopic uterine vasculopathy may lead to abortion or stillbirths of virus-positive fetuses in mid and late pregnancy. Additionally, the induction of cytokines and hormones in the local environment during pregnancy may accelerate the adhesion of PRV-infected monocytes to ECs, further contributing to miscarriage in sows (29).

The gE gene deletion-engineered vaccines are widely used to immunize commercial pig herds and wildlife against PRV. Since 2011, outbreaks of PR caused by emerging PRV variants have occurred in Chinese pig herds immunized with the Bartha-K61 strain. The classical PRV attenuated vaccines have been demonstrated to provide incomplete protection for pigs (34, 35). Scientists have conducted research and developed genetically engineered vaccines against the novel 2011 PR. Currently, only two vaccine types have been licensed: a genetically modified inactivated vaccine against the PRV HeN1201 strain (2019) and a natural four-gene deletion (gI/gE/Us9/Us2) vaccine against the PRV C strain (2017) (36, 37). The active ingredients of certain herbs have also been demonstrated to act as PRV inhibitors. For example, resveratrol (trans-3,4,5-trihydroxystilbene; Res) has been shown to possess immunomodulatory, anti-inflammatory, and antiviral activities (38) and has been observed to protect rotavirus-infected piglets by reducing inflammatory responses and enhancing immune function (39).

#### 3.1.3 Porcine reproductive and respiratory syndrome

Porcine reproductive and respiratory syndrome (PRRS), also referred to as porcine blue ear disease, is caused by the porcine reproductive and respiratory syndrome virus (PRRSV). Infected sows exhibit reproductive disorders, which are primarily manifest in abortion, mummified fetus, weak fetuses and stillbirths. During the late gestation period, the abortion rate can exceed 30.0%, and the piglets exhibit severe respiratory disorders, with a mortality rate of 35.0% ~ 40.0%. Infected sows can be detoxified through excretion in feces, saliva, milk, and so forth, but the detoxification cycle is lengthy (40).

**Figure 3:**
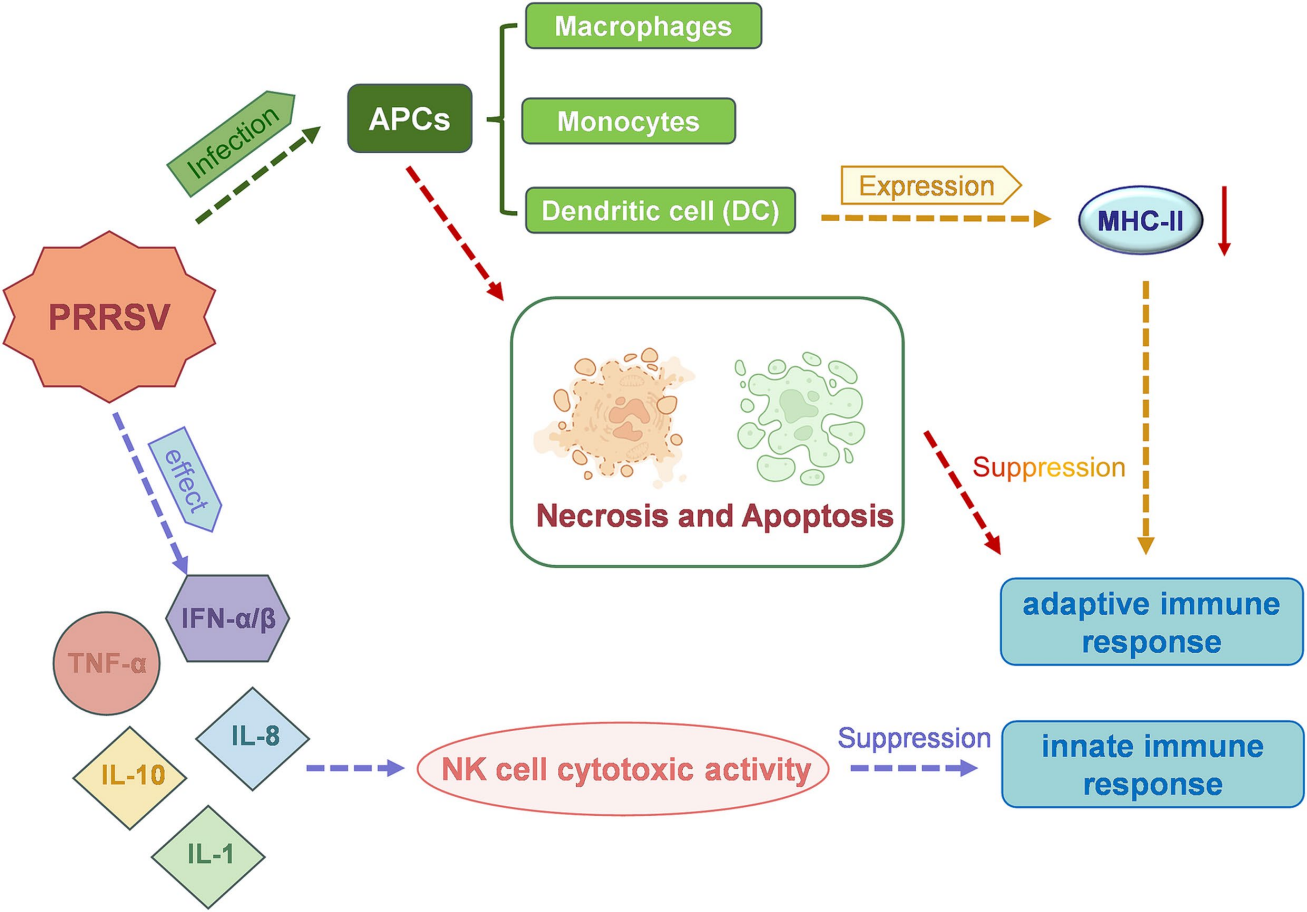
A schematic representation of the mechanism of action of porcine reproductive and respiratory syndrome virus (PRRSV), which leads to reproductive dysfunction in sows.

PRRSV primarily infects macrophages and cells of the monocyte lineage, including dendritic cells (DCs) (40). Infection in a breeding pig results in significantly reduced immunity, leading to the development of mixed and secondary infections, further exacerbating the disease severity. PRRSV can modulate various inflammatory cytokines (Figure 3), including interferon-α (IFN-α), tumor necrosis factor-α (TNF-α), as well as interleukins such as IL-1, IL-8 and IL-10, to regulate the host innate immune response (41). PRRSV infection also reduces the expression of major histocompatibility complex (MHC) class II molecules on the surface of antigen-presenting cells. Additionally, the virus has been shown to induce the death of host cells through both apoptotic and necrotic mechanisms, thus inhibiting the functions of DCs and evading the host’s adaptive immune response (42, 43). It is also possible that PRRSV may reach the endometrial connective tissue by infecting endometrial vascular migrating mononuclear cells. Viral replication leads to local cellular infection and peripheral cell death, which in turn causes fetal detachment from the placenta or cellular degeneration, ultimately causing fetal death (44). Furthermore, PRRSV infection may result in inflammatory damage to the endometrium, placenta, blood vessels, and myometrium of pregnant sows. This may reduce the intensity of vascular endothelial growth factor (VEGF) immunostaining, which could affect cell proliferation at the maternal-fetal interface and submucosal angiogenesis and impact fetal viability (45, 46).

The primary objective of controlling PRRS is to prevent infection, establish optimal herd immunity, and minimize the risk of infection, which is a systematic process. PRRS vaccines can be broadly classified into live attenuated and inactivated vaccines. Two categories of live attenuated vaccines exist: those derived from classical strains and those derived from highly pathogenic strains (47). Given that PRRSV is an RNA virus, its high variability and rapid evolution pose significant challenges for the design and development of PRRS vaccines. Currently, attenuated applications are widely employed but face challenges such as revertant mutations, virulence enhancement, and strain recombination. Precise knowledge of the antibody titer of PRRS can ascertain the existence and severity of the disease, determine the immune status of the herd, and inform the improvement of the immunization strategy as needed, thereby reducing the clinical infection rate of PRRS and gradually achieving the goal of disease purification.

#### 3.1.4 Japanese encephalitis B

Epidemic encephalitis B is a zoonotic infection caused by the mosquito-borne Japanese encephalitis virus (JEV), which targets the central nervous system of both humans and animals. In its natural habitat, JEV primarily infects humans and animals via the “pig-mosquito-human” cycle. Pigs serve as “amplifying hosts” and represent the largest reservoir, multiplier and disperser of the virus. The virus can multiply in large quantities in pigs, resulting in overt viremia (48). JEV viral particles proliferate primarily in tissues, including connective tissue, skeletal muscle, cardiac muscle, smooth muscle, lymphoreticular tissue, and endocrine and exocrine glands, among others. The virus can also cross the blood–brain barrier to access the central nervous system, infecting neuronal cells. The pro-inflammatory and chemotactic factors released from the infected neuronal cells can activate microglia to produce more inflammatory factors, leading to an “inflammatory storm” in the central nervous system, which ultimately causes viral encephalitis and massive neuronal death (49, 50). Infection with encephalitis B in pigs is largely asymptomatic, although it can cause several other clinical signs, including high fever in fattening pigs, abortion in pregnant sows, stillbirth, mummified fetuses, and premature or delayed delivery, among other symptoms. It can also cause acute inflammation of the testes in boars, resulting in enlarged testes on one or both sides, followed by atrophy, hardening, and, ultimately, the loss of breeding capacity (51).

Vaccination has been demonstrated to provide a beneficial protective effect on infectious diseases such as Japanese encephalitis (JE), which are zoonotic and transmitted by insect vectors. However, it is not feasible to eradicate these diseases through vaccination alone. The three major types of vaccine currently in use worldwide are the inactivated mouse brain vaccine, the inactivated cellular vaccine, and the live attenuated encephalitis vaccine. The inactivated mouse brain vaccine is the most widely produced and used vaccine and is the only inactivated Japanese encephalitis B vaccine approved by the World Health Organization (WHO) and commercialized for human use (52). Therefore, it is necessary to implement a comprehensive strategy that includes industrial structure adjustment, mosquito control, intermediate host prevention, and final host immunization to effectively control JE epidemics.

#### 3.1.5 Porcine circovirus diseases

Porcine circovirus (PCV) is a single-stranded circular DNA virus with four identified genotypes (PCV1 - PCV4) (53). PCV2 is the predominant genotype associated with postweaning multisystemic wasting syndrome (PMWS) and reproductive disorders in sows (54). PCV2 infection in sows can result in increased rates of return to estrus, abortion, and stillbirths. Furthermore, PCV2 can be vertically transmitted from the mother to the fetus, causing myocarditis and interstitial pneumonia. In severe cases, fetal mummification and death may occur (55). Epidemiological surveys conducted in Italy (2013–2018) revealed a rising prevalence of PCV2d detection in domestic pigs, with a similar trend observed in wild boars (56).

PCV2 can bind to cellular receptors via its capsid protein. Given the diversity of viral attachment receptors, PCV2 has the ability to infect multiple tissues and organs in pigs. Studies have shown that the likelihood of PCV2 infection varies among different pig breeds, indicating that pig genetics can influence the infectivity of PCV2 in the host (57). PCV2 can penetrate mature oocytes through a compromised zona pellucida and has the capacity to reduce the developmental competence of oocytes (58). In embryos with compromised zona pellucida, PCV2 infection significantly reduces survival rates, with only 6.4% of infected embryos surviving compared to 65.4% of negative controls (59).

Panax notoginseng saponins and arctigenin (ACT) can alleviate oxidative stress in mice infected with PCV2, thereby partially suppressing viral replication (60, 61). These findings offer novel therapeutic perspectives for PCV diseases (PCVD). Commercial PCV2 vaccines currently available include inactivated vaccines (Fostera^™^ PCV, Circovac^®^) and subunit vaccines (Porcilis^®^ PCV, Circumvent^®^, Ingelvac CircoFLEX^®^) (62).

#### 3.1.6 Classical swine fever

Classical Swine Fever (CSF), an acute and highly contagious disease caused by Classical Swine Fever Virus (CSFV), poses a significant threat to pig health and the swine industry. CSFV, a single-stranded RNA virus, belongs to the genus Pestivirus within the family Flaviviridae (63).

CSF is endemic in regions of Central and South America, Eastern Europe, Asia, and Africa. While the prevalence of highly virulent CSFV strains has diminished in recent years, infections caused by moderately virulent strains persist. Morbidity can reach 100%, while mortality rates fluctuate according to viral strain virulence (64).

The effects of CSFV on sow reproduction are highly dependent on the gestational stage at which infection occurs. Early gestation infections may cause abortions, stillbirths, or fetal mummification. In contrast, infections during mid-to-late gestation that result in the live birth of persistently infected piglets can induce neurological disorders and growth retardation. CSFV exhibits immunosuppressive properties, causing a significant reduction in white blood cells in infected pigs, with apoptosis primarily occurring in the thymus, spleen, lymph nodes, and bone marrow (65). Moreover, CSFV can inhibit the host’s antiviral response through activation of the IL-10-STAT1 pathway (66).

Vaccination is a crucial strategy for CSF prevention. However, inactivated whole virus vaccines are neither effective nor available. Live attenuated vaccines (LAV) are extensively used in CSF-endemic regions but cannot distinguish between natural infection and vaccination. Conversely, the E2 subunit vaccine (Porcilis® Pesti) and the chimeric virus vaccine (Suvaxyn CSF Marker) have DIVA (differentiation of infected from vaccinated animals) capabilities, making them appropriate for settings where such differentiation is necessary (67).

### 3.2 Bacterial infections

#### 3.2.1 Brucellosis

Brucellosis is a zoonotic infection caused by the bacterium *Brucella* spp. (68), with approximately 500,000 new cases resulting from animal-to-human transmission occurring globally each year (69). The prevalence of *Brucella* in pig herds has been reported worldwide, with the infection rate in Europe being 17.4% (70). In the European Union, North America and Australia, the prevalence of *Brucella suis* (*B. suis*) in domestic pigs is lower due to the implementation of eradication programs. However, the risk of pathogen reintroduction in wild pigs persists (71), as shown by the higher prevalence in feral pigs (15.0%) than in domestic pigs (1.1%).

*B. suis* is currently subdivided into five biovars, with the primary biovars responsible for brucellosis in pigs being biovars 1, 2, and 3 (71). In regions outside of Europe, the main causative agents of swine brucellosis are biovars 1 and 3 (72), whereas in Europe, pigs are mainly infected with biovars 2 (73). *Brucella* is a facultative intracellular parasitic bacterium capable of evading the host’s innate and adaptive immune responses (74) and resistant to some antibiotics, thereby causing a characteristic pathological manifestation in the infected host. Cellular immunity plays a major role in eradication of the intercellular infection, while serum antibodies can only act against extracellular *Brucella* spp. Consequently, the immunity produced by immunization with inactivated vaccines is markedly weak. Currently, live attenuated vaccines are the most commonly used worldwide for preventing and controlling swine brucellosis. These include the live *B. suis*. Vaccine (S2 strain) developed in China, and the live *B. abortus* vaccine (SRB51 strain) developed in the United States (75, 76). These vaccines can be administered orally, subcutaneously, or intramuscularly to pigs. However, these live vaccines are inadequate for protecting swine against *B. suis* infection and pose a risk of infection to humans. Furthermore, it is difficult to differentiate between vaccine immunity and natural infection. At present, there are no commercially available vaccines for protecting domestic or feral swine against *B. suis* infection. Although not a feasible solution in all situations, whole-herd depopulation is the most effective regulatory mechanism for controlling swine brucellosis (71).

#### 3.2.2 Listeriosis

Listeriosis is a sporadic infectious disease of humans, livestock, and poultry caused by *Listeria monocytogenes*. The Centers for Disease Control and Prevention (CDC) estimates that there are approximately 1,600 infection cases and 260 deaths related to the disease annually (77). *L. monocytogenes* can invade various eukaryotic cells, including epithelial cells, fibroblasts and macrophages, among others (78) and disseminate to the placenta, fetus, and neonates, with approximately 14% of clinically confirmed cases occurring during pregnancy. In pigs, infection with *L. monocytogenes* is primarily associated with the development of meningitis, septicemia, and mononucleosis, as well as abortion in pregnant sows.

Once it has entered enterocytes, *L. monocytogenes* spreads throughout the body and subsequently crosses the placental and the blood–brain barriers, entering phagocytic and non-phagocytic epithelial cells and proliferating within these cells. Access to specialized phagocytic cells, such as macrophages, is a passive process, and active entry into non-phagocytic cells, such as intestinal cells, fibroblasts, endothelial cells, hepatocytes, and epithelial cells, necessitates the presence of two surface proteins, InlA and InlB (79). There is currently no effective vaccine available to prevent this disease. Treatment with antibiotics is usually needed for the control of the infection caused by Listeriosis (80). The administration of high doses of streptomycin, penicillin, gentamicin, and sulfonamides in pigs at the initial stages of the disease can result in favorable therapeutic outcomes. Nevertheless, treatment of suckling pigs with neurological symptoms often proves ineffective (81). In addition, certain Listeria strains have demonstrated resistance to commonly employed antibiotics (penicillin, gentamicin, and sulfonamides), complicating future control and treatment efforts (82).

#### 3.2.3 Chlamydiosis

*Chlamydia* is a febrile, chronic, and contact infectious disease caused by *Chlamydia* infection in pigs. Four species of *Chlamydia* can infect pigs: *Chlamydia suis*, *C. psittaci* (Cps), *C. abortus*, and *C. pecorum* (Cpe). The most prevalent form of cross-infection is between *C. suis* and *C. abortus* (83). Pregnant sows infected with *Chlamydia* tend to be asymptomatic, and the disease occurs most frequently in primiparous sows, with abortion rates ranging from 40.0 to 90.0%.

The pathogenic mechanisms of *C. abortus* and *C. suis* remain unknown. The infectious elementary body (EB) enters cells to form phagosomes, and *Chlamydia*’s major outer membrane protein family (MOMP) prevents phagosomes from fusing with lysosomes, thus facilitating the replication of *Chlamydia* within the phagosome and the destruction of host cells. Additionally, *Chlamydia* can produce endotoxin-like substances analogous to those produced by Gram-negative bacteria. These substances inhibit host cell metabolism and directly destroy host cells. The infected organism elicits a delayed hypersensitivity reaction, which results in immunopathological damage to tissue cells. Following infection, *C. abortus* induces the production of cytokines, including IFN-*γ*, TNF-α, IL-4, and IL-10, which can alter the infected cells and result in miscarriage (84). A recent study has demonstrated that host animals infected with *C. abortus* exhibit gut microbial dysbiosis, which may also contribute to abortion in animals (85).

*Chlamydia* is a multisymptomatic contact zoonosis that represents a significant public health concern. The implementation of an efficacious vaccination program has the potential to mitigate the morbidity and post-illness severity observed in animal populations while also serving to impede the further regional dissemination of *C. abortus* and the emergence of antibiotic resistance. Currently, commercial vaccines for swine chlamydiosis are not widely used in the pig industry and are largely confined to laboratory development and preclinical trials. The CPAF protein of *Chlamydia trachomatis* has recently been shown to be highly immunogenic in pigs (86). An experimental subunit vaccine targeting *C. abortus* has demonstrated protective immunity in piglets (87). However, the commercial *C. abortus* 1B vaccine strain for ruminants (Cevac® Chlamydia, Ceva Animal Health Ltd.) may still induce abortions in vaccinated animals, potentially facilitating the spread of *C. abortus* (88).

#### 3.2.4 Leptospirosis

*Leptospira* spp. are spiral-shaped bacteria capable of surviving in diverse environments, particularly in warm and humid conditions (89). These bacteria can infect a wide range of animals, including pigs, and cause various diseases. Transmission occurs through contact with contaminated urine, water, or soil (90). In pigs, the most important serovars associated with reproductive issues include Bratislava, Pomona, and Tarassovi (91). These serovars can induce lesions in the uterus and placenta, impairing fertilization and embryo implantation, ultimately resulting in infertility or reduced conception rates (92, 93).

*Leptospira* spp. can rapidly enter the bloodstream, causing leptospirosis bacteremia, which induces a robust inflammatory response, leading to tissue damage and organ dysfunction. Additionally, these bacteria can evade the host immune system, resulting in persistent damage. Regular vaccination can effectively reduce the incidence of leptospirosis (94). Furthermore, enhancing the hygiene management of pig pens and preventing contact with contaminated water and soil can significantly lower the risk of transmission (95).

#### 3.2.5 Campylobacteriosis

Campylobacteriosis, caused by *Campylobacter* spp., can significantly affect the reproductive system of sows, with the specific manifestations and severity varying depending on the bacterial strain and the host’s immune status.

*Campylobacter* is a genus of Gram-negative, spiral-shaped bacteria that are highly motile and obligate microaerophilic (96). The most common species associated with swine are *Campylobacter coli* and *Campylobacter jejuni* (97, 98). Although, these bacteria primarily colonize the gastrointestinal tract of pigs, their potential to cause reproductive disorders remains less well-documented than their effects on the digestive system.

In sows, *Campylobacter* infection can cause reproductive tract inflammation, including endometritis and cervicitis (99). Such inflammation can disrupt normal reproductive processes, leading to early embryonic loss, reduced fertility, and prolonged inter-estrus intervals (100). However, the specific pathological changes in the reproductive system of sows due to *Campylobacter* infection are less well-documented compared to those in cattle.

The pathogenicity of *Campylobacter* spp. is attributed to several virulence factors, including their ability to adhere to and invade host cells, produce toxins such as cytolethal distending toxin (CDT), and evade the host immune system (101, 102). Their spiral shape and motility facilitate penetration and colonization of mucosal surfaces, including those of the reproductive tract (103). In the context of reproductive disorders, these bacteria can trigger immune responses that cause inflammation and tissue damage, ultimately impairing reproductive function.

Preventing and controlling *Campylobacter* infections in swine requires a comprehensive approach. Regular monitoring of pig herds for *Campylobacter* presence facilitates early detection and management of infections. In cases of clinical infection, appropriate antibiotic treatment is essential; however, it should be used judiciously to prevent the development of antibiotic-resistant strains.

#### 3.2.6 Swine erysipelas

*Erysipelothrix rhusiopathiae*, a Gram-positive bacterium, is the primary etiological agent of swine erysipelas (SE) and can adversely affect sow reproductive performance. The bacterium comprises multiple serotypes, among which types 1a, 1b, and 2 are predominant in causing disease in pigs (104).

Surface proteins of *E. rhusiopathiae*, including SpaA, promote bacterial adhesion to host cells and recruit host plasminogen, thereby enhancing pathogenicity (105). The pathogenesis of *E. rhusiopathiae* in sows involves its capacity to induce systemic infections or septicemia, causing inflammation and tissue damage in multiple organs, including the reproductive system (106). The bacterium disseminates through the bloodstream, inducing lesions in the placenta and fetal tissues, which can lead to fetal death and abortion (107). Chronic infections may also result in endocarditis and arthritis, further impairing the sow’s overall health and reproductive performance (108).

Vaccination is essential for controlling erysipelas, with live attenuated vaccines or bacterins being commonly used (109). Pre-farrowing vaccination of sows boosts maternal antibody levels in piglets, offering enhanced protection against the disease.

### 3.3 Parasitic infections

Toxoplasmosi*s* is a common zoonotic protozoan disease caused by *Toxoplasma gondii*, a parasite that infects animals, including pigs, which is prevalent in China and the United States (110). *T. gondii* is present in both domestic and wild pigs, with a global prevalence of *T. gondii* infection in domestic pigs being as high as 30.0% (111, 112). A national survey of boars in the United States (2022) revealed a seropositivity rate of *T. gondii* of approximately 27.0% (113). A study (2024) conducted in Italy investigating the prevalence of *T. gondii* IgG positivity in 174 wild boar meat juices collected from forest and peri-urban environments revealed a rate of 22.6% (114). Infection of pregnant sows with *T. gondii* may result in the transmission of the parasite to the fetus via the placenta, potentially leading to abortion, stillbirth, malformation of the fetus, or underdevelopment of the piglet (115). *Toxoplasma* cysts can form and persist in the body for a considerable period of time, rendering them difficult to eliminate. Combining sulfonamides with antimicrobial adjuncts has been demonstrated to be a more efficacious treatment. The definitive host of *T. gondii*, the cat, excretes feces containing infectious oocysts, which, when ingested by pigs, can lead to infection. Therefore, the most effective method of preventing toxoplasmosis in pigs is implementing efficient management strategies for cats.

Developing a toxoplasmosis vaccine is a challenging endeavor, primarily due to the intricate life history of *T. gondii*, the numerous infection routes, and the formation of cysts to evade the immune response of the host. No commercial vaccine for toxoplasmosis is currently available; however, two vaccine groups developed by the National Veterinary Quarantine Institute of Korea have been reported to be effective in preventing toxoplasmosis (116).

## 4 Integrated management measures

**Figure 4:**
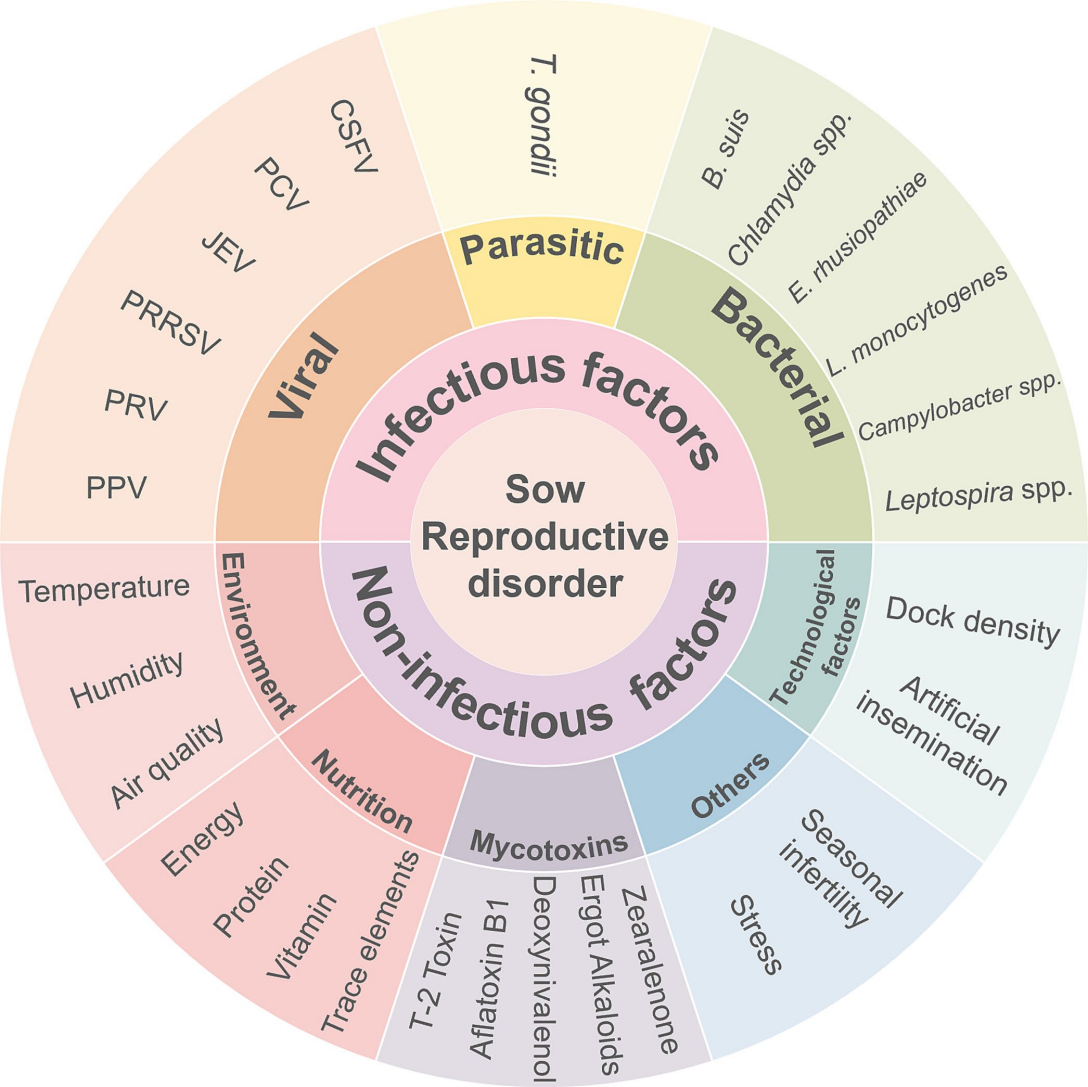
The main causes of reproductive disorders in sows.

The occurrence of reproductive disorders in sows is attributable to a combination of single and superimposed factors (Figure 4). According to the biosecurity protocols for pig farms, introducing pigs from external sources should be kept to a minimum. It is recommended that non-potable water sources within the farm, including streams, ponds, and open drainage ditches, be treated and disinfected regularly to control the spread of diseases through water (116), as they may contain pathogenic organisms such as *Leptospira*. Additionally, the prompt removal of manure and feed residues, the maintenance of optimal ventilation within pig houses, and the implementation of appropriate nutritional balance can collectively enhance the pigs’ resistance to disease.

Vaccination represents one of the most efficient and cost-effective methods currently available to prevent reproductive disorders in pigs (Table 3). It is important to consider several factors when developing a comprehensive vaccination program, including the presence of maternal antibodies in sows, the onset age of the disease in pigs, and the season of occurrence. The use of antimicrobial medications for preventive purposes entails a shift in their application from treatment to prevention, thereby reducing the probability of bacterial disease occurrence. Regular blood tests and fecal examinations are recommended for parasitic eggs to ensure proper internal and external parasite control. Furthermore, pigs exhibiting low antibody levels should be promptly administered with booster vaccinations.

**Table 3:** Commercial vaccine developed against infectious pathogens involved in sow reproductive disorder.

Pathogen	Vaccine name	Type of vaccine	Marketing company	References
PPV	Porcilis^©^ Parvo	Inactivated	MSD Animal Health	Vereecke et al. (144)
	ReproCyc^®^ ParvoFLEX	Subunit	Boehringer Ingelheim Vetmedica	Garcia-Morante et al. (145)
	ERYSENG^®^ PARVO	Bivalent	HIPRA	Sánchez-Matamoros et al. (47)
	BIOSUIS ParvoEry	Inactivated	Bioveta	European Medicines Agency (146)
PRV	Suvaxyn Aujeszky 783 + O/W	Live attenuated	Zoetis	European Medicines Agency (146)
	AUSKIPRA^®^ BK	Inactivated	HIPRA	Aznar et al. (147)
	AUSKIPRA^®^ GN	Live attenuated	HIPRA	Álvarez et al. (148)
	Ingelvac^®^ Aujeszky MLV	Live attenuated	Boehringer Ingelheim Vetmedica	Kondibaeva et al. (149)
PRRSV	Suvaxyn PRRS MLV	Live attenuated	Zoetis	Kreutzmann et al. (150)
	Porcilis^©^ PRRS	Live attenuated	Intervet International BV	Barna et al. (151)
	Porcilis PRRS	Live attenuated	MSD Animal Health	Stadler et al. (152)
	UNISTRAIN^®^ PRRS	Live attenuated	HIPRA	Sánchez-Matamoros et al. (47)
	Ingelvac PRRSFLEX EU	Live attenuated	Boehringer Ingelheim Vetmedica	Kraft et al. (153)
	ReproCyc PRRS EU	Live attenuated	Boehringer Ingelheim Vetmedica	
JEV	IXIARO^®^	Inactivated	Valneva Austria	Jelinek et al. (154)
CSFV	Suvaxyn^®^ CSF Marker	Viral vector	Zoetis	Panyasing et al. (155)
	Porcilis^®^ pesti	Subunit	MSD Animal Health	Coronado et al. (156)
	Bayovac^®^ CSF Marker	Subunit	Bayer, Leverkusen	
	TWJ-E2^®^	Subunit	–	Gong et al. (157)
PCV2	Porcilis^®^ PCV ID	Subunit	Intervet International BV	Puig et al. (158)
	MHYOSPHERE^®^ PCV ID	Inactivated	HIPRA	
	Ingelvac CircoFLEX^®^	Subunit	Boehringer Ingelheim Vetmedica	
	Fostera™PCV	Inactivated	Pfizer Animal Health	Afghah et al. (62)
	Circovac^®^	Inactivated	CEVA-PHYLAXIA	Guo et al. (159)
	Porcilis^®^ PCV	Subunit	Intervet International BV	
	Suvaxyn Circo^®^	Inactivated recombinant chimeric	Zoetis	Tameling et al. (160)
	CircoMax^®^	Inactivated recombinant chimeric	Zoetis	Venegas-Vargas et al. (161)
*E. rhusiopathiae*	ERYSENG^®^ PARVO	Bivalent	HIPRA	Sánchez-Matamoros et al. (47)
	Eryseng^®^	Bacterin	HIPRA	Sanchez-Tarifa et al. (109)
	Suvaxyn^®^ E-Oral	Live attenuated	Zoetis	
	Nobilis^®^ Erysipelas	Bacterin	MSD Animal Health	
	Ruvax^®^	Lysate bacterin	Boehringer Ingelheim Vetmedica	Opriessnig et al. (162)
*Leptospira*	Porcilis^®^ Ery + Parvo + Lepto	Inactivated	MSD Animal Health	Mascher et al. (163)

Other disease vectors, such as rodents, reportedly transmit several bacterial diseases, including salmonellosis, swine erysipelas and leptospirosis (117), as well as several viral diseases, including parvovirus and Japanese encephalitis virus. In addition, insects such as mosquitoes and flies act as vectors for several diseases. It is, therefore, imperative to maintain high hygiene standards and implement effective pest control measures to eradicate insects and rodents within the farm.

## 5 Conclusions and future prospects

Reproductive disorders in sows have always been a major risk factor for pig production, especially those caused by malignant, infectious and zoonotic diseases. It is necessary to monitor the zoonotic pathogens that cause reproductive disorders in sows and understand their interactions with both humans and animal hosts. It is also necessary to consider the effects of environmental perturbations, while implementing the One Health concept to achieve a holistic vision of the health of breeding sows. This concept encompasses not only the pigs, but also the interrelationships between humans, pigs, and other organisms within the agricultural ecosystem. This approach aims to comprehensively understand the health status of all biological organisms on large-scale farms. Therefore, the One Health concept is not only concerned with preventing health crises in pigs, but also closely related to maintaining health, environmental quality and nutritional standards in animal feed. It is reasonable to deduce that the health of both humans and pigs can be enhanced through the One Health approach.

The success of the One Health concept relies on collaborative efforts across multiple sectors, including human and veterinary medicine, as well as environmental and wildlife health. This collaborative approach will help to reduce and prevent future zoonotic disease outbreaks.

## Glossary

CPI: Consumer price index
SMEDI: Stillbirths, mummification, embryonic death, and infertility
PPV: Porcine parvovirus
PRV: Pseudorabies virus
Cps: *Chlamydophila psittaci*
ROS: Reactive oxygen species
StAR: Steroidogenic acute regulatory protein
3β-HSD: 3β-hydroxysteroid dehydrogenase
P450scc: P450 cholesterol side-chain cleavage enzyme
PR: Porcine pseudorabies
EC: Endothelial cell
PRRS: Porcine respiratory and reproductive syndrome
PRRSV: Porcine respiratory and reproductive syndrome virus
DCs: Dendritic cells
IFN-α: interferon-α
TNF-α: Tumor necrosis factor-α
MHC: Major histocompatibility complex
VEGF: Vascular endothelial growth factor
APCs: Antigen presenting cells
JEV: Japanese encephalitis virus
JE: Japanese encephalitis
WHO: World Health Organization
PCV: Porcine circovirus
ACT: Arctigenin
CSF: Classical swine fever
CSFV: Classical swine fever virus
LAV: Live attenuated vaccines
CDT: Cytolethal distending toxin
SE: Swine erysipelas
*B. suis*: *Brucella suis*
CDC: Centers for disease control and prevention
Cpe: *Chlamydia pecorum*
*C. suis*: *Chlamydia suis*
*C. abortus*: *Chlamydia abortus*
EB: Elementary body
MOMP: Major outer membrane protein family
*T. gondii*: *Toxoplasma gondii*
PCR: Polymerase chain reaction
ELISA: Enzyme-linked immunosorbent assay
LAT: Latex agglutination test
IHC: Immunohistonchemistry
FAVN: Fluorescent antibody virus neutralization
MAT: Microscopic agglutination test
